# COVID-19 Vaccine Booster Hesitancy (VBH) among Healthcare Professionals of Pakistan, a Nationwide Survey

**DOI:** 10.3390/vaccines10101736

**Published:** 2022-10-17

**Authors:** Muhammad Subhan Arshad, Imran Masood, Imran Imran, Hamid Saeed, Imran Ahmad, Iqra Ishaq, Hafsa Yaseen, Muqarrab Akbar, Muhammad Omer Chaudhry, Muhammad Fawad Rasool

**Affiliations:** 1 Department of Pharmacy Practice, Faculty of Pharmacy, Bahauddin Zakariya University, Multan 60800, Pakistan; 2 Department of Pharmacy, Southern Punjab Institute of Health Sciences, Multan 60000, Pakistan; 3 Department of Pharmacy, The Islamia University of Bahawalpur, Bahawalpur 63100, Pakistan; 4 Department of Pharmacology, Faculty of Pharmacy, Bahauddin Zakariya University, Multan 60800, Pakistan; 5 University College of Pharmacy, Allama Iqbal Campus, University of the Punjab, Lahore 54000, Pakistan; 6 Department of Political Science, Bahauddin Zakariya University, Multan 60800, Pakistan; 7 School of Economics, Bahauddin Zakariya University, Multan 60800, Pakistan

**Keywords:** COVID-19 vaccines, booster dose, vaccine hesitancy, vaccine acceptance, Pakistan

## Abstract

Background: The waning vaccine immunity and emergence of new variants of SARS-CoV-2 led health authorities across the globe to administer booster doses (BDs) of the COVID-19 vaccine. Hence, the current study aimed to assess the COVID-19 vaccine booster hesitancy (VBH) amongst Pakistani healthcare professionals (HCPs). Methods: A nationwide survey-based study was carried out from April 2022 to May 2022. The online self-administered questionnaire was utilized to collect data regarding demographics (age, gender, marital status, profession, residential area, and province), COVID-19 infection history (infection history, onset, and clinical severity of disease), previous COVID-19 vaccination (type of vaccination and the number of doses), attitudes towards BDs (acceptance, rejection, and hesitancy), and psychological drivers of VBH (perceived effectiveness, vaccine safety, risk/benefit ratio, and vaccine type preference). We assessed the association between the dependent variable attitudes of study participants, regarding BDs and independent variables (demographics, COVID-19 infection history, previous COVID-19 vaccination, and psychological drivers of VBH), by using the Chi-square test/Fisher exact test. Results: Among the 1164 study participants, 51.4% were male, and 80.4% were medical professionals. The half of study participants (52.1%) agreed to take the COVID-19 vaccine BD or had already taken it, while the rest of them refused (34.7%) or hesitated (24.2%) to take it. These attitudes of the participants were significantly associated (*p* < 0.001) with psychological divers about the COVID-19 vaccine BD. Conclusion: This study revealed that Pakistani HCPs hesitant to take the COVID-19 vaccine BD had concerns about the safety, efficacy, and risk/benefits ratio of the vaccine’s BD. To eliminate the hesitancy, regarding BD in HCPs, certain educational strategies should be implemented by health authorities to address the concerns of HCPs.

## 1. Introduction

Coronavirus disease 2019 (COVID-19) has caused significant morbidity and mortality all around the globe, since its emergence in late 2019 [1]. As of April 2022, worldwide, more than 505 million people been infected by COVID-19, and among them, 6.2 million died of it [2]. Mass vaccination against COVID-19 played a key role in protecting against the risk of infection and the severity of the disease to minimize the burden of the disease [3,4,5]. To cope with the emerging new variants and decline in humoral immunity over time, health authorities started to administer booster doses (BDs) of COVID-19 vaccines to enhance the protection of vaccinated people, as revealed by different studies [6,7,8].

Vaccine hesitancy is one of the major threats to global health, as a hurdle in herd immunity to protect people by vaccination [9]. The World Health Organization (WHO) has defined it as a delay in acceptance or refusal to be vaccinated, despite the accessibility of vaccination facilities [10]. Misinformation and conspiracy theories lead to vaccine hesitancy against the COVID-19 vaccination program, especially in the early phase [11,12,13]. The reported acceptance rate of the COVID-19 vaccine ranges from 23% to 97%, with the lowest rate from the Middle East [14]. The acceptance rate of the COVID-19 vaccine among the Pakistani population was 48% to 72%, reported in different studies [11,15,16,17].

Healthcare professionals (HCPs) are a high-risk population, due to the nature of their work, so they were prioritized to get vaccinated, preferably even for the administration of the BDs [18]. Therefore, achieving a high vaccination rate among HCPs is crucial, as the first group to be vaccinated and a most trusted source of guidance regarding health issues, so HCPs could be the source of credible local experiences as role models for the rest of the community [19]. The prevalence of COVID-19 vaccine booster hesitancy (VBH) among HCPs is comparable to the general population across different countries [20,21,22,23,24,25]. As of April 2022, among 121 million fully vaccinated individuals only 6.9 million received the COVID-19 vaccine BD [26]. Therefore, we aimed to assess the COVID-19 VBH and the attitude of the HCPs towards BDs in Pakistan. Furthermore, the secondary objective was to determine the association between independent variables (demographics, COVID-19 infection history, vaccination history, and psychological drivers) with attitude towards BD and the potential predictors of vaccine acceptance among the targeted population.

## 2. Materials and Methods

### 2.1. Study Design, Population, and Sample Size

A web-based, self-administered questionnaire (SAQ) was used to collect data under a cross-sectional study design from April 2022 to May 2022. The HCPs currently living in Pakistan and aged more than 18 years were included in the study. The strengthening the reporting of observational studies in epidemiology (STROBE) guidelines were followed to report this cross-sectional study [27]. A minimum sample size of 1068 was calculated through an online sample size calculator (Rao soft), with a 3% margin of error, 95% confidence interval, 50% response distribution, and targeted population size of 485,277, based on a report by the Pakistan Bureau of Statistics [28], which was further inflated by 10% (N = 107) to counteract any errors in the completion of SAQ, resulting in a final sample size of 1175. 

### 2.2. Questionnaire 

A pre-validated SAQ from the previous study (among the HCPs of Czechia, regarding COVID-19 VBH) was utilized to prepare the draft SAQ [20]. That SAQ comprised five sections, with a total of 19 close-ended questions. The first section comprised questions regarding demographic data of the participants, i.e., age, gender (male/female), marital status (married/single), profession (physician/dentist/pharmacist/nurse/supportive staff), residential area (rural/urban), and province (Punjab/Sindh/Baluchistan/KPK/Capital Islamabad/Gilgit Baltistan). The second section included questions about COVID-19 vaccination history (yes/no), type of vaccination, the number of doses (one/two/three), and side effects experienced after vaccination (yes/no). While the questions about COVID-19 infection history (yes/no/maybe), onset (before 1st dose/between doses/after 2nd dose), and clinical severity of disease (mild/moderate/severe/critical) were included in the third section. In the fourth section, participants were asked about their willingness to take the COVID-19 vaccine’s BD (yes/no/maybe). The fifth section comprised the possible psychological drivers of COVID-19 vaccine BD acceptance, i.e., (a) perceived effectiveness: preventing severe illness, symptomatic infection, community transmission, and mutations, (b) perceived safety: equally safe as previous doses and seriousness of side effects, (c) perceived risk/benefit ratio and prioritization to take BD, and (d) vaccine previous dose satisfaction, vaccine selectivity, and vaccine type preference. 

The expert panel of subject specialists, related to pharmacy practice and public health, assured the content validity of the SAQ. To confirm the face validity and reliability of the SAQ, a pilot study was performed in the targeted population, with a sample size of 30. The SAQ was found reliable, with a Cronbach alpha’s value of 0.8.

### 2.3. Data Collection 

A snowball technique was utilized to collect data from the targeted population (HCPs) by designing an online version of SAQ via Google Forms (Google LLC, Menlo Park, CA, USA), whose link was distributed through several platforms, such as WhatsApp, Facebook, E-mail, etc. The checklist for reporting results of internet E-surveys (CHERRIES) guidelines were followed for online survey conduction and data collection [29]. 

### 2.4. Ethics

Electronic informed consent was acquired from every participant before the administration of the study questionnaire. To avoid the Hawthorne bias, no data were collected in the study that could reveal the identity of participants, to ensure their privacy. The current study was approved by the ethics committee of the Department of Pharmacy Practice, Faculty of Pharmacy, Bahauddin Zakariya University, Multan (reference No. Acad/PRAC/22/03). This study was conducted under the Declaration of Helsinki. 

### 2.5. Statistical Analysis

The statistical analyses were performed by utilizing the statistical package for the social sciences (SPSS), version 21.0 (IBM, Armonk, NY, USA). To present all the study variables, descriptive statistics were utilized, whereas categorical variables were presented as frequencies (n) and percentages (%). To test the association between independent variables (demographics, COVID-19 vaccination history, COVID-19 infection history, psychological drivers) and the dependent variable of attitude towards BD, inferential analyses were performed utilizing the Chi-square test (χ2). Fisher’s exact test was used for a test with a predicted cell count of less than 5. After that, univariate logistic regression analysis was performed to identify possible predictors of vaccine for each significant independent variable in χ2 analysis. The proposed psychological drivers were analyzed through multivariate regression analysis, after being adjusted for gender, marital status, residential area, vaccine type taken, vaccination side effects, and infection history. A significance level of ≤0.05 was considered statistically significant, and a confidence level of 95% was assumed during all the inferential statistics.

## 3. Results

Among the total 1190 respondents, 26 responses were excluded from the final analysis, due to incompleteness, which led to a completeness rate of 97.8%. Out of 1164 study participants, 598 (51.4%) were male, and 566 (48.6%) were female, with a mean age of 28.79 (SD ± 5.01) years. Amongst the study participants, 831 (71.5%) were unmarried, and 936 (80.4%) belonged to a medical profession (physician, dentist, and pharmacist). Among them, 821 (71.0%) were from the province of Punjab, and 880 (75.6%) were living in urban areas. The demographic details of the survey participants are presented in Table 1.

Among the study participants, only 272 (23.4%) reported being infected by COVID-19 (confirmed by test). Amongst those who were infected by COVID-19, 55.9% were infected before the first dose of vaccination, and 66.9% reported mild severity of the disease. A total of 304 (26.1%) participants reported their previous infection status as “maybe” because they never tested for it. There was a statistically significant difference (*p* < 0.001) in reported COVID-19 infection history across gender, as females (30.4%) were most commonly infected, as compared to males (16.7%). The onset and severity of the disease had a significant association with the profession, gender, and age groups of the participants, details can be seen in Table 2.

The majority of the participants (95.7%) were vaccinated against COVID-19. Most of them received two doses (82.8%), and the rest received either three doses (9.9%) or one dose (7.4%). According to participants reporting, the most commonly administered vaccine is the inactivated virus vaccine (75.4%), followed by the mRNA vaccine (15.4%). Among those vaccinated participants, only 18% reported that they had experienced any side effects after vaccination. There were significant gender- (*p* = 0.008) and age-based (*p* < 0.001) differences in the type of vaccine administered (Table 3). Medical professionals (11.2%), females (11.2%), and participants aged ≥ 30 years (13.8%) significantly received the third dose of COVID-19 vaccine, as compared to allied health professionals (4.6%), male (8.7%), and aged less than 30 (8.0%).

Out of 1164 study participants, 52.1% reported their acceptance (willing to take BD/already taken it) to take the COVID-19 vaccine BD, while 34.7% rejected it, and 24.2% hesitated to take it. The participant attitudes towards the COVID-19 booster had significant difference across the demographic variables, i.e., gender (*p* < 0.001), marital status (*p* = 0.045), and residential area (*p* < 0.001). The COVID-19 disease-related history (being infected (*p* < 0.001), onset (*p* < 0.001), and severity of illness (*p* < 0.001)) of the study participants was also significantly associated with the vaccination attitudes of the study participants. The vaccine acceptance (66.2%) was highest among the previously infected persons. None of the respondents with critically and severely ill conditions during the previous infection hesitated to take a BD of COVID-19 vaccine. The attitude regarding the BD of the COVID-19 vaccine had significant differences among the determinants of vaccination history (vaccine taken (*p* < 0.001), type of vaccine (*p* < 0.001), number of doses (*p* < 0.001), and experience of side effects (*p* < 0.001)) of the study participants. More than half of the participants (53.8%) who did not experience any side effects during previous doses of the vaccine accepted taking a BD. The BD acceptance was also highest among the participants who have taken the inactivated virus vaccines (80.9%), followed by the mRNA vaccines (15.6%), further details can be seen in the Table 4. 

The study participants reported their views regarding various drivers related to COVID-19 vaccine BD on a three-point Likert scale (agree, neutral, and disagree). Among the study participants, 50.3%, 49.5%, and 47.8% agreed about protection against severe illness, symptomatic infection, and community transmission by administration of BD. Half of the participants (50.2%) agreed that currently available BDs are safer than previous doses, and more than half of the participants (54.6%) worried about side effects due to vaccination, as compared to previous doses. In response to the driver regarding perceived susceptibility, 53.8% of participants agreed that a BD’s benefits outweighs its risk, and 52.9% agreed to receive a BD on priority bases. The majority of participants (69.7%) preferred to take the mRNA vaccines as a BD, followed by the inactivated virus vaccines (24.7%). All the psychological drivers were also significantly associated (*p* < 0.001) with the attitude of the study participants toward the BD of the COVID-19 vaccine. The higher levels of BD acceptance (72.4%, 75.7%, and 68.7%) were present among the participants having an agreement with the perceived effectiveness of BD for severe illness, symptomatic infection, and community transmission, respectively. Likewise, participants who agreed with the perceived safety of the BD have a higher level of BD acceptance (74.3%), as compared to others having disagreement (29.4%) with that statement. The detailed psychological determinants of COVID-19 vaccine BD-related attitudes of the study participants can be seen in Table 5. The BD accepting (76.6%), rejecting (55.8%), and hesitant (53.2%) groups of HCPs are stated to promote the mRNA vaccines, such as BD.

The significantly associated independent variables in the Chi-square test were included in the univariate regression analysis to assess the possible predictors of vaccine acceptance. The female Pakistani HCPs were 1.270 (1.009–1.599) more likely to be accepting of the COVID-19 vaccine BD, as compared to males and married Pakistani HCPs, which were 1.385 (1.071–1.790) times more expected to accept BD, as compared to unmarried. The participants who had previously taken the inactivated virus vaccine and mRNA vaccine were 5.109 (3.076–7.982) and 4.500 (2.537–7.982) times more likely to be willing to take the BD, as compared to those who had taken the adenovirus vector vaccine previously (Table 6).

The proposed psychological drivers of COVID-19 vaccine BD acceptance were assessed by using multivariate logistic regression analysis, while adjusting for significant independent variables in univariate analysis. The participants who had an agreement with the ability of BD to control severe illness and symptomatic infection were 3.181 (2.004–5.050) and 2.770 (1.812–4.234) more likely to be accepting BD, as compared to their counterparts. The participants who agreed with an equal safety profile of BD as that of the primer doses and had satisfaction regarding the previous vaccine types administered to them were 4.220 (2.814–6.329) and 2.144 (1.431–3.213) more likely to be willing to obtain a BD of the COVID-19 vaccine (Table 7). 

## 4. Discussion

The BD of the COVID-19 vaccine became essential, due to the reported decrease in the immunity among the vaccinated population [30,31]; hence, the topic of current research became more crucial to be assessed. To the best of our knowledge, this study is a pioneer study evaluating the hesitancy against COVID-19 vaccine BD among healthcare professionals in Pakistan. The current study has shown that more than half of the study participants (52.1%) reported acceptance of a BD of the COVID-19 vaccine, while 34.7% rejected taking the BD. Among the study participants, 24.2% reported hesitancy to take a BD of the COVID-19 vaccine. A study among HCPs of Czech reported a higher level of COVID-19 vaccine BD acceptance (71.3%) [20], while the US HCPs have shown even more acceptance (92.1%) to take BDs COVID-19 vaccine [23]. The reported acceptance of BDs among the public of the Pakistani population ranged from 77.8% to 89.4% [32,33]. The participants of the current study that were infected by COVID-19, even after getting primer vaccine doses, could be the reason for the low acceptance of the BDs of the vaccine.

Approximately one-fourth of the study participants (23.4%) reported being infected by COVID-19 previously, and the majority of the participants (55.9%) were infected with COVID-19 before the 1st dose of vaccine, which is consistent with other studies from Pakistan [32,33]. Most of the participants (95.7%) were vaccinated against COVID-19, and the furthermost commonly administered vaccine type was the inactivated virus vaccine (66.2%), while a similar rate of vaccination (97%) was reported among the general population of Pakistan [32]. The vaccine rejection and hesitancy were more commonly seen among the participants who had taken the adenovirus vector vaccine previously, as compared to mRNA and inactivated virus vaccines, which is consistent with previous studies [20,24]. That could be due to the low efficacy and more intense side effects of the adenovirus vaccines, in comparison to mRNA vaccines and inactivated virus vaccines [34]. 

Almost half of the participants (47.8% to 54.6%) agreed about the perceived effectiveness, safety, and risk/benefit ratio of the COVID-19 vaccine’ BD, while a similar percentage of the participants (52.1%) accepted the BD of the COVID-19 vaccine. A prior study in Czech also revealed a similar ratio between BD acceptance and the psychological drivers of COVID-19 vaccine’s BD among the HCPs [20]. All the psychological drivers were significantly associated (*p* < 0.001) with the attitude of the participants towards vaccine BD (acceptance, rejection, or hesitancy), which was consistent with the results of a survey from Czechian HCPs [17]. It can be concluded from the similarity in the results of these studies from the different geographical regions that promoting the agreement on psychological drivers could reduce the hesitancy regarding the COVID-19 vaccine. Most of the participants reported the mRNA vaccine to be promoted for the booster dose, compared to our previous study, that was conducted at the time of the COVID-19 vaccine’s approval in Pakistan, which could be related to the circulation of conspiracy beliefs regarding the COVID-19 vaccine at that time [11]. 

There are some limitations in the current study. The study participants may not be the true representative of all Pakistani HCPs, as a snowball sampling technique was utilized, due to which, the results cannot be generalized. The SAQ was in the English language and circulated through the internet, so HCPs with limited English proficiency and limited internet availability may not have been able to participate in the study. The majority of participants were from the province of Punjab, which may have influenced the extrapolation of the results. The possible predictors, such as severeCOVID-19 experiences of relatives, which were not included in the current study, may be considered a limitation of the study. 

## 5. Conclusions

Under the umbrella of study limitations, only half of the Pakistani HCPs (52.1%) accepted the BD of the COVID-19 vaccine, whereas 24.2% were still hesitant, and 34.7% were against the administration of BDs. The perceived effectiveness (against severe illness and symptomatic infection), perceived safety (equally safe as primer dose, the occurrence of side effects), perceived risk–benefit ratio, self-prioritization, satisfaction with the previously administered vaccine, and selectivity of BD were found to be robust predictors for the acceptance of the BD, while effectiveness against community transmission and mutation control were not important for the targeted population. The policymakers and the health administration should take the necessary steps to enhance the agreement of HCPs with psychological drivers of COVID-19 vaccine BD through evidence-based educational approaches, such as seminars, educational videos, and by supplying of latest clinical literature, regarding vaccines, for the reduction of VBH.

## Figures and Tables

**Table 1:** Demographic details of the respondents (N = 1164).

Variables	Outcomes	Frequency	Percentage
Age Groups	<30 Years	772	66.3%
	≥30 Years	392	33.7%
Gender	Male	598	51.4%
	Female	566	48.6%
Marital Status	Single	832	71.5%
	Married	332	28.5%
Profession	Medical Professionals	936	80.4%
	Allied Health Professionals	228	19.6%
Residential Area	Urban	880	75.6%
	Rural	284	24.4%
Province	Punjab	826	71.0%
	Sindh	143	12.3%
	Baluchistan	72	6.2%
	Capital Islamabad	61	5.2%
	AJK/Gilgit-Baltistan	62	5.3%

**Table 2:** COVID-19-related history of the respondents.

Variable	Outcomes	Overall (N = 1164)	Medical Professionals (N = 936)	Allied Health Professionals (N = 228)	*Sig.*	Male (N = 598)	Female (N = 566)	*Sig.*	<30 Years (N = 772)	≥30 Years (N = 392)	*Sig.*
Infected by SARS-CoV-2 (COVID-19)	Yes ^¶^	272 (23.4%)	218 (23.3%)	54 (23.7%)	**0.001**	100 (16.7%)	172 (30.4%)	**<0.001**	172 (22.3%)	100 (25.5%)	0.17
	No	588 (50.5%)	494 (52.8%)	94 (41.2%)		304 (50.8%)	284 (50.2%)		386 (50.0%)	202 (51.5%)	
	Maybe	304 (26.1%)	224 (23.9%)	80 (35.1%)		194 (32.4%)	110 (19.4%)		214 (27.7%)	90 (23.0%)	
Onset of infection ^¶^	Before 1st dose	152 (55.9%)	130 (59.6%)	22 (40.7%)	0.004 *	40 (40%)	112 (65.1%)	<0.001 *	82 (47.7%)	70 (70.0%)	<0.001 *
	Between doses	10 (03.7%)	10 (04.6%)	0 (00.0%)		0 (0.0%)	10 (5.8%)		10 (5.8%)	0 (0.0%)	
	After 2nd dose	110 (40.4%)	78 (35.8%)	32 (59.3%)		60 (60.0%)	50 (29.1%)		80 (46.5%)	30 (30.0%)	
Severity of infection ^¶^	Mild	182 (66.9%)	140 (64.2%)	42 (77.8%)	0.002 *	60 (60.0%)	112 (65.1%)	<0.001 *	112 (65.1%)	70 (70.0%)	<0.001 *
	Moderate	50 (18.4%)	48 (22.0%)	2 (03.7%)		70 (70.0%)	40 (23.3%)		50 (29.1%)	0 (0.0%)	
	Severe	30 (11.0%)	20 (09.2%)	10 (18.5%)		10 (10.0%)	20 (11.6%)		10 (5.8%)	20 (20.0%)	
	Critical	10 (03.7%)	10 (04.6%)	0 (00.0%)		(10.0%)	0 (0.0%)		0 (0.0%)	10 (10.0%)	

Bold fonts indicate the statistically significant results (*p* ≤ 0.05), ^¶^ Participants previously infected by COVID-19, * Fisher exact test used.

**Table 3:** COVID-19 vaccine-related details of the respondents.

Variable	Outcomes	Overall (N = 1164)	Medical Professionals (N = 936)	Allied Health Professionals (N = 228)	*Sig.*	Male (N = 598)	Female (N = 566)	*Sig.*	<30 Years (N = 772)	≥30 Years (N = 392)	*Sig.*
COVID-19 vaccine received	Yes ^†^	1114 (95.7%)	896 (95.7%)	218 (95.6%)	0.940	578 (96.7%)	536 (94.7%)	0.1	752 (97.4%)	362 (92.3%)	**<0.001**
	No	50 (4.3%)	40 (4.3%)	10 (4.4%)		20 (3.3%)	30 (5.3%)		20 (02.6%)	30 (07.7%)	
Vaccine type taken ^†^	mRNA vaccines	172 (15.4%)	142 (15.8%)	30 (13.8%)	0.679	82 (14.2%)	90 (16.8%)	**0.008**	80 (10.6%)	92 (254%)	**<0.001**
	Adenovirus vector vaccines	102 (9.2%)	80 (8.9%)	22 (10.1%)		40 (06.9%)	62 (11.6%)		62 (8.2%)	40 (11.0%)	
	Inactivated virus vaccines	840 (75.4%)	674 (75.2%)	166 (76.1%)		456 (78.9%)	384 (71.6%)		610 (81.1%)	230 (63.5%)	
Doses taken ^†^	Single dose	82 (7.4%)	72 (8.0%)	10 (4.6%)	**0.002**	62 (10.7%)	20 (3.7%)	**<0.001**	50 (06.6%)	32 (8.8%)	**0.003**
	Two doses	922 (82.8%)	724 (80.8%)	198 (90.8%)		466 (80.6%)	456 (85.1%)		642 (85.4%)	280 (77.3%)	
	Three doses	110 (9.9%)	100 (11.2%)	10 (4.6%)		50 (8.7%)	60 (11.2%)		60 (08.0%)	50 (13.8%)	
Experienced side effects after vaccination ^†^	Yes	200 (18.0%)	128 (14.3%)	72 (33.0%)	<0.001 *	80 (13.8%)	120 (22.4%)	<0.001 *	150 (19.9%)	50 (13.8%)	**0.003**
	No	904 (81.1%)	768 (85.7%)	136 (62.4%)		488 (84.4%)	416 (77.6%)		592 (78.7%)	312 (86.2%)	
	Maybe	10 (0.9%)	0 (0.0%)	10 (4.6%)		10 (1.7%)	0 (0.0%)		10 (1.3%)	0 (0.0%)	

Bold fonts indicate the statistically significant results (*p* ≤ 0.05), ^†^ Participants vaccinated against COVID-19, * Fisher exact test used.

**Table 4:** The association of demographics, COVID-19 disease, and vaccination history towards attitudes of participants, regarding booster dose of COVID-19 vaccine.

Variable		Outcomes	Acceptance N = 606		Rejection N = 276		Hesitancy N = 282		*Sig.*
			N	%age	N	%age	N	%age	
Demographics	Gender	Male	294	49.2%	174	29.1%	130	21.7%	<0.001
		Female	312	55.1%	102	18.0%	152	26.9%	
	Age	<30 Years	406	52.6%	174	22.5%	192	24.9%	0.398
		≥30 Years	200	51.0%	102	26.0%	90	23.0%	
	Marital status	Single	414	49.8%	206	24.8%	212	25.5%	0.045
		Married	192	57.8%	70	21.1%	70	21.1%	
	Profession	Medical professionals	476	50.9%	232	24.8%	228	24.4%	0.157
		Allied Health Professionals	130	57.0%	44	19.3%	54	23.7%	
	Residential area	Urban	526	59.8%	152	17.3%	202	23.0%	<0.001
		Rural	80	28.2%	124	43.7%	80	28.2%	
COVID-19 Vaccination history	COVID-19 vaccine received	Yes ^†^	576	51.7%	256	23.0%	282	25.3%	**<0.001**
		No	30	60.0%	20	40.0%	0	0.0%	
	Vaccine type taken ^†^	mRNA vaccine	90	15.6%	52	20.3%	30	10.6%	**<0.001**
		Adenovirus vector vaccines	20	3.5%	40	15.6%	42	14.9%	
		Inactivated virus vaccine	466	80.9%	164	64.1%	210	74.5%	
	Doses taken ^†^	Single dose	40	48.8%	22	26.8%	20	24.4%	<0.001 *
		Two doses	436	47.3%	234	25.4%	252	27.3%	
		Three doses	100	90.9%	0	0.0%	10	9.1%	
	Experienced side effects after vaccination ^†^	Yes	90	45.0%	50	25.0%	60	30.0%	<0.001 *
		No	486	53.8%	206	22.8%	212	23.5%	
		Maybe	0	0.0%	0	0.0%	10	100.0%	
COVID-19 Infection History	Infected by SARS-CoV-2 (COVID-19)	Yes ^¶^	180	66.2%	42	15.4%	50	18.4%	**<0.001**
		No	264	44.9%	172	29.3%	152	25.9%	
		Maybe	162	53.3%	62	20.4%	80	26.3%	
	Onset	Before 1st dose	100	65.8%	32	21.1%	20	13.2%	<0.001 *
		Between doses	10	100.0%	0	0.0%	0	0.0%	
		After 2nd dose	70	63.6%	10	9.1%	30	27.3%	
	Severity of infection	Mild	110	60.4%	32	17.6%	40	22.0%	<0.001 *
		Moderate	40	80.0%	0	0.0%	10	20.0%	
		Severe	20	66.7%	10	33.3%	0	0.0%	
		Critical	10	100.0%	0	0.0%	0	0.0%	

Bold fonts indicate the statistically significant results (*p* ≤ 0.05), ^†^ Participants vaccinated against COVID-19, ^¶^ participants previously infected by COVID-19, * Fisher exact test used.

**Table 5:** The association of psychological determinants of COVID-19 vaccine booster dose with vaccination attitudes of participants.

Variables	Outcomes	Overall		Acceptance N = 606		Rejection N = 276		Hesitancy N = 282		*Sig.*
		N	%age	N	%age	N	%age	N	%age	
Vaccine’s booster dose will protect against severe COVID-19 infection (severe illness).	Agree	586	50.3%	424	72.4%	70	11.9%	92	15.7%	**<0.001**
	Neutral	486	41.8%	162	33.3%	144	29.6%	180	37.0%	
	Disagree	92	7.9%	20	21.7%	62	67.4%	10	10.9%	
Vaccine’s booster dose will protect from symptoms of COVID-19 infection (symptomatic infection).	Agree	576	49.5%	436	75.7%	50	8.7%	90	15.6%	**<0.001**
	Neutral	432	37.1%	140	32.4%	130	30.1%	162	37.5%	
	Disagree	156	13.4%	30	19.2%	96	61.5%	30	19.2%	
Booster doses of the COVID-19 vaccine will prevent transmission of SARS-CoV-2 and its variants in the community (community transmission).	Agree	556	47.8%	382	68.7%	82	14.7%	92	16.5%	**<0.001**
	Neutral	476	40.9%	174	36.6%	132	27.7%	170	35.7%	
	Disagree	132	11.3%	50	37.9%	62	47.0%	20	15.2%	
Booster doses are similarly safe as were previous doses of COVID-19 vaccines (equal safety).	Agree	584	50.2%	434	74.3%	90	15.4%	60	10.3%	**<0.001**
	Neutral	478	41.1%	142	29.7%	144	30.1%	192	40.2%	
	Disagree	102	8.8%	30	29.4%	42	41.2%	30	29.4%	
Administration of booster doses will produce more severe side effects, in comparison to the previous dose (inferior safety).	Agree	636	54.6%	486	76.4%	90	14.2%	60	9.4%	**<0.001**
	Neutral	414	35.6%	90	21.7%	134	32.4%	190	45.9%	
	Disagree	114	9.8%	30	26.3%	52	45.6%	32	28.1%	
The benefits of the COVID-19 vaccine’s booster dose are greater than its risks (risk─benefit ratio).	Agree	626	53.8%	394	62.9%	102	16.3%	130	20.8%	**<0.001**
	Neutral	406	34.9%	172	42.4%	124	30.5%	110	27.1%	
	Disagree	132	11.3%	40	30.3%	50	37.9%	42	31.8%	
I will receive the COVID-19 vaccine’s booster dose on priority bases (self-priority).	Agree	616	52.9%	422	68.5%	94	15.3%	100	16.2%	**<0.001**
	Neutral	418	35.9%	164	39.2%	102	24.4%	152	36.4%	
	Disagree	130	11.2%	20	15.4%	80	61.5%	30	23.1%	
I will prefer to take the COVID-19 vaccine’s booster dose after evidence-based confirmation of its capability to prevent circulating new variants of SARS-CoV-2 (mutation control).	Agree	406	34.9%	142	35.0%	164	40.4%	100	24.6%	**<0.001**
	Neutral	426	36.6%	234	54.9%	82	19.2%	110	25.8%	
	Disagree	332	28.5%	230	69.3%	30	9.0%	72	21.7%	
I will prefer to take a different type/brand of vaccine as a booster dose from earlier doses (vaccine satisifaction).	Agree	352	30.2%	230	65.3%	52	14.8%	70	19.9%	**<0.001**
	Neutral	568	48.8%	244	43.0%	152	26.8%	172	30.3%	
	Disagree	244	21.0%	132	54.1%	72	29.5%	40	16.4%	
Certain COVID-19 vaccine types/brands should be purchased by the government to administer as a booster dose (vaccine selectivity).	Agree	454	39.0%	282	62.1%	70	15.4%	102	22.5%	**<0.001**
	Neutral	496	42.6%	212	42.7%	134	27.0%	150	30.2%	
	Disagree	214	18.4%	112	52.3%	72	33.6%	30	14.0%	
Which COVID-19 vaccine type should be promoted as a booster dose (preferred vaccine).	mRNA Vaccines	768	69.7%	464	76.6%	154	55.8%	150	53.2%	**<0.001**
	Adenovirus vector vaccines	62	5.6%	10	01.7%	42	15.2%	72	25.5%	
	Inactivated virus vaccines	272	24.7%	132	21.8%	80	29.0%	60	21.3%	

Bold fonts indicate the statistically significant results (*p* ≤ 0.05).

**Table 6:** Univariate regression analysis of the predictors of COVID-19 vaccine BD acceptance among study participants.

Predictors	B	SE	Wald	OR (CI 95%)	*Sig.*
Gender (female vs. male)	0.239	0.118	4.133	1.270 (1.009–1.599)	**0.042**
Marital status (married vs. single)	0.325	0.131	6.173	1.385 (1.071–1.790)	**0.013**
Residential area (urban vs. rural)	1.332	0.149	80.192	3.789 (2.831–5.072)	**<0.001**
Vaccinated (yes vs. no)	0.337	0.295	1.308	0.714 (0.400–1.272)	0.253
Vaccine type taken (mRNA vaccine vs. adenovirus vector vaccines)	1.504	0.292	23.459	4.500 (2.537–7.982)	**<0.001**
Vaccine type taken (inactivated virus vaccine vs. adenovirus vector vaccines)	1.631	0.259	39.691	5.109 (3.076–7.982)	**<0.001**
Side effects after vaccination (no vs. yes)	0.328	0.157	4.368	1.388 (1.021–1.887)	**0.037**
Previously infected (yes vs. no)	0.761	0.145	27.678	2.140 (1.612–2.842)	**<0.001**

Bold fonts indicate the statistically significant results (*p* ≤ 0.05).

**Table 7:** Multivariate logistic regression analysis of proposed psychological drivers for acceptance of COVID-19 vaccine BD among the healthcare professionals of Pakistan.

Predictors	B	SE	Wald	OR (CI 95%)	*Sig.*
Severe Illness: Agree	1.157	0.236	24.099	3.181 (2.004–5.050)	**<0.001**
Symptomatic Infection: Agree	1.019	0.217	22.142	2.770 (1.812–4.234)	**<0.001**
Community Transmission: Agree	−0.259	0.226	1.307	0.772 (0.496–1.203)	0.253
Equal Safety: Agree	1.440	0.207	48.514	4.220 (2.814–6.329)	**<0.001**
Inferior Safety: Disagree	−0.858	0.322	7.100	0.424 (0.225–0.797)	**0.008**
Risk Benefit Ratio: Agree	−1.329	0.275	23.351	0.265 (0.155–0.454)	**<0.001**
Self-Priority: Agree	1.062	0.253	17.598	2.891 (1.761–4.748)	**<0.001**
Mutation Control: Disagree	0.066	0.209	0.100	1.068 (0.709–1.610)	0.752
Vaccine Satisfaction: Agree	0.763	0.206	13.672	2.144 (1.431–3.213)	**<0.001**
Vaccine Selectivity: Agree	−0.482	0.201	5.752	0.617 (0.416–0.916)	**0.016**

Bold fonts indicate the statistically significant results (*p* ≤ 0.05).

## Data Availability

The data that support the findings of this study are available from the corresponding author upon reasonable request.
